# Epidemiology, clinical features, and surgical outcomes of acute acquired concomitant esotropia associated with myopia

**DOI:** 10.1371/journal.pone.0280968

**Published:** 2023-05-18

**Authors:** Matilde Roda, Natalie di Geronimo, Nicola Valsecchi, Lorenzo Gardini, Michela Fresina, Aldo Vagge, Luigi Fontana, Costantino Schiavi

**Affiliations:** 1 Ophthalmology Unit, DIMEC, University of Bologna, Bologna, Italy; 2 IRCCS Azienda Ospedaliero-Universitaria di Bologna, Bologna, Italy; 3 DINOGMI, Polyclinic Hospital San Martino IRCCS, University Eye Clinic, Genoa, Italy; Cairo University Kasr Alainy Faculty of Medicine, EGYPT

## Abstract

**Purpose:**

To analyze epidemiology, clinical features, and surgical outcomes of type III acute acquired concomitant esotropia (Bielschowsky esotropia (BE)).

**Methods:**

The medical charts of patients diagnosed with acquired concomitant esotropia between 2013 and 2021 were reviewed. Assessed data were age, gender, age at diplopia onset, age at the diagnosis, refraction, visual acuity, neuroimaging, diplopia onset, angle of deviation, stereopsis, surgical procedure, amount of surgery, and relapse of diplopia after surgery. Moreover, we investigated the correlation between the use of electronic devices and the onset of diplopia.

**Results:**

One hundred seventeen patients (mean age 35.07 ± 15.81 years) were included in the study. The mean delay to the diagnosis was 3.29 ± 3.62 years. Myopia range was 0 to 17 diopters spherical equivalent. 66,3% spent more than 4 hours a day using laptops, tablets, or smartphones at the onset of diplopia, and 90,6% presented a subacute onset. None showed neurologic signs or symptoms. Patients who underwent surgery were ninety-three, with a rate of surgical success of 93.6%, and a relapse rate of 17.2%. A negative correlation resulted between pre-operative deviation and age at diagnosis (ρ = -0.261; p<0.05), whereas factors associated with surgical failure were older age at diplopia onset (p = 0.042) and longer delay between onset and diagnosis (p = 0.002).

**Conclusion:**

We registered an outstanding increase in prevalence of BE, which could be related to the exponential increase in the use of electronic devices for professional, educational, and recreational purposes. A prompt diagnosis and an augmented dose of surgery allows good motor and sensory results.

## Introduction

Acute acquired concomitant esotropia (AACE) is a relatively rare subtype of esotropia. Typically, it is characterized by late-onset, minimal accommodative mechanism and good binocular function [1]. In 1958, Burian and Miller introduced the classification of this disease into three types [2]. Type III AACE, also known as Bielschowsky esotropia (BE), was described first by Von Graefe in 1864 and then by Bielschowsky in 1922 as a sudden onset esotropia with diplopia characterized by an inner deviation at distance and maintenance of fusion at near, no evidence of paralysis, conserved binocular fusion and associated with myopia of 5 diopters (D) or less. Hoyt and Good modified this definition and underlined the relationship with various degrees of myopia, and the constant and equal deviation at both near and distance fixations [3]. Although the aetiology is still not wholly understood, it has been recently associated with excess close work in adults, which could produce an imbalance of the convergence and divergence tone of the extraocular muscle [4]. The increasing diffusion of electronic devices (pc, tablets, smartphones) in our daily activities increased the number of hours people spend on near work. It seems to have contributed to the increase in the prevalence of this type of strabismus in the last years, particularly during the Covid-19 pandemic [5–7].

This study aimed to determine the epidemiology and clinical features of patients with BE who presented to our department in the last decade. Moreover, we tried to investigate environmental factors that contribute to the development of the strabismus and to analyze possible etiopathogenetic mechanisms.

## Material and methods

This was a retrospective, longitudinal, single-centre study involving patients who presented at the Sant’Orsola-Malpighi Ophthalmology Department of Bologna (Italy) between January 2013 and October 2021 with the diagnosis of AACE. The study was performed in accordance with the principles of the Declaration of Helsinki and was approved by the local Ethics Committee (code: 775/2022/Oss/AOUBo). All patients gave written informed consent.

Inclusion criteria were: type III AACE diagnosis according to Burian-Miller classification modified by Hoyt and Good; no history of ocular trauma; no history of previous strabismus surgery; no history of amblyopia; no history of ocular, neurologic or systemic diseases which can interfere with ocular alignment; any known cause of interruption of binocular fusion mechanisms.

Assessed data were age, gender, occupation, age at diplopia onset, age at the diagnosis, neuroimaging, best corrected visual acuity (BCVA), refractive error in cycloplegia, power spectacles of myopia, deviation for distance and near fixation, stereopsis assessed with TNO stereotest (reported as absent or present), surgical procedure, relapse of diplopia after surgery. Additional information was obtained from questionnaires which included the type of diplopia onset (“acute” or “subacute”), the type of near visual activity, and the daily use of electronic devices (such as laptops, tablets and smartphones) at the onset of diplopia. We classified the time spent on electronic devices reported by the patients as: a) <1 hour, b) 1–4 hours, c) 4–8 hours, and d) >8 hours.

The deviation angle was measured using Cover Test with prisms in a highly dissociative way, inviting the patient to fix a small accommodative stimulus at distance (5 m) and at near (33 cm) alternately in order to stimulate accommodative convergence and to unmask the total amount of the angle of deviation. The measurement was done after a minimum of one month of wearing full myopic correction and with and without glasses.

In the case of surgery, the procedure consisted of a bilateral symmetric recession of medial rectus muscles performed by two surgeons (C.S. and M.F.) under general anaesthesia. Surgical success was defined by the resolution of diplopia and a post-operative deviation <10 PD at distance and at near. In addition, we evaluated the relapse rate as the number of patients presenting a new onset of diplopia or a deviation angle > 10 PD at distance and at near after surgery.

Statistical analysis was performed using IBM Statistical Package for Social Sciences (SPSS) software, version 26. The normality of values has been evaluated with the Shapiro-Wilk test, and non-parametric tests have been used. We estimated medians and interquartile intervals of considered variables. Spearman test has been used to correlate age at diplopia onset and diagnosis delay to the deviation for distance and near fixation in preoperatory assessment. The Mann-Whitney test has been used to evaluate the differences between patients exposed to electronic devices for less and more than 4 hours and to assess different characteristics between patients with surgery success and failure. The Mann-Whitney test was also used to evaluate the characteristics of patients who developed a relapse during follow-up and those who did not. Finally, linear regression analysis has been used to assess the correlation between recession extent, considered a dependent variable (y), and angle deviation at distance, considered an independent variable (x. The formula for the simple linear regression is: y = a + bX. The “a” is the intercepta and the “b” is the regression coefficient. We regarded as statistically significant a *p-value* <0,05.

## Results

**Fig 1:**
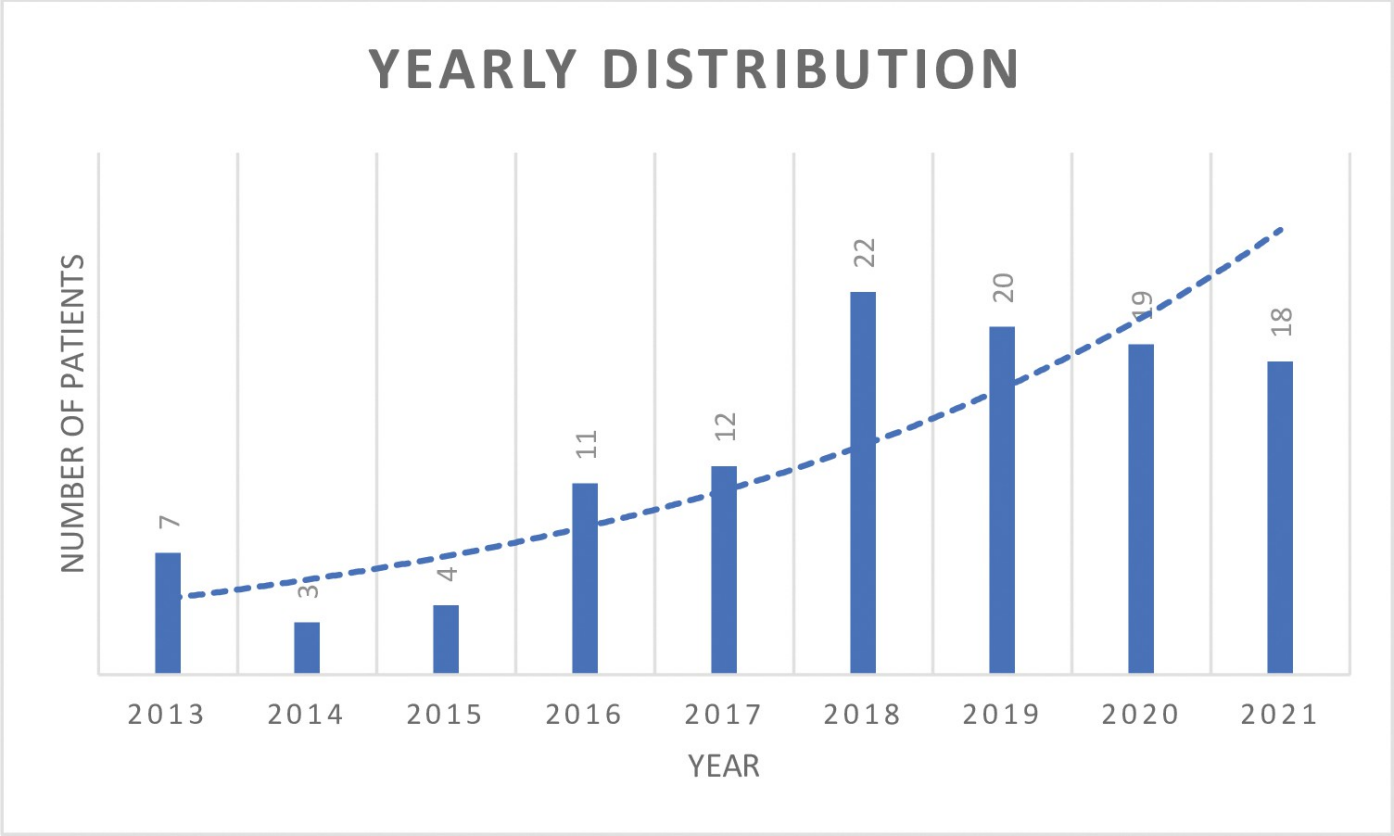
Yearly distribution of Bielschowsky esotropia.

A total of 117 patients (78 males and 39 females) with a diagnosis of type III AACE were included in the study. The yearly distribution is shown in Fig 1. Mean age at diplopia onset was 35.07 ± 15.81 years (range: 11 to 74 y), with a mean delay to the diagnosis of 3.29 ± 3.62 years (range: 0.5 to 18.09). Mean spherical equivalent was -4.60 ± 2.80 D (range: 0 to -15D) in the right eye and -4.55 ± 2.85 D (range: 0 to -17D) in the left eye. Two patients showed unilateral myopia, 37 (31.6%) had low myopia (<3D), 47 (40.2%) had moderate myopia (3 to 6D), and 31 (26.5%) had high myopia (>6D). Moreover, 11 patients underwent surgical correction of refractive error, and seven patients were bilaterally pseudophakic. Biomicroscopy of the anterior segment, fundus examination and ocular motility were normal in each patient. The mean deviation angle was 27.03 ± 7.96 PD for distance and 26.54 ± 8.33PD for near fixation. The deviation angle did not change significantly with or without full refractive correction (p>0.05).

All patients performed a brain Computed Tomography (CT) or Magnetic Resonance Imaging (MRI) at the onset of diplopia, which showed: no pathological findings in 92.5% of cases, undefined pathological findings in 6% and inactive demyelinating lesions in 0.5%. No abnormalities of the extra-ocular muscles were observed. In this case, lesions did not explain the development of diplopia. In patients with myopia >10D, we requested orbital TC/MRI to exclude characteristics of heavy eye syndrome, such as the rupture of the band between the superior rectus muscle and lateral rectus muscle, or the dislocation of these muscles in medial and inferior direction, respectively.

Ninety-two patients (78,6%) answered the questionnaire. Patients with a history of sudden development of diplopia (“acute onset”) were 10 (9,4%), whereas patients with a history of intermittent diplopia (“subacute onset”) were 82 (90,6%). Sixty-one patients (66,3%) spent more than 4 hours daily using laptops, tablets, or smartphones. Of these patients, 75,3% reported that the prolonged use of such devices (>4 hours daily) was for an occupational purpose. There was no statistically significant difference between the clinical features of patients who wore glasses at near and patients who did not (p>0.05).

Patients who underwent surgery were 93, with a surgical success rate of 93,6%. We performed a bilateral symmetric recession of medial rectus muscles with fixed sutures. The surgical dose was decided according to the preoperative characteristics of the patient, in particular the angle deviation, fusional convergence amplitudes, ocular motility, and refractive error. Mean recession performed was 6,3 ± 1,04 mm. Comparing the extent of our recession with the surgical recession dosage proposed by Parks, we highlight that the dose was increased by 33.1% ± 8.3%. The mean post-op follow-up was 2,4 ± 1,2 years and the relapse rate, evaluated as the number of patients presenting a new onset of diplopia or a deviation angle > 10PD at distance and at near after surgery, was 17,2%. We did not register surgical complications.

The other 24 patients are still on the waiting list.

Clinical features are summarized in Table 1.

**Table 1:** Clinical features of included patients.

**Clinical features**	
**Age at diplopia onset (y)**	35,07 ± 15,81 (9 to 71)
**Age at diagnosis (y)**	38,36 ± 16,44 (11 to 74)
**Interval between onset and diagnosis (y)**	3,29 ± 3,62 (0,5 to 18,09)
**Sex (M/F) (no.)**	78/39
**Spherical equialent (D)**	
OD	-4,62 ± 2,76 (0 to -15)
OS	-4,55 ± 2,85 (0 to -17)
**Deviation at the diagnosis (PD)**	
Distance fixation	27,03 ± 7,96 (10 to 50)
Near fixation	26,46 ± 8,33 (10 to 50)
**Onset of diplopia**	
Subacute/acute	106/11
Distance/near	110/7
**Stereopsis (y/n)**	110/7
**Use of electronic devices (h)**	
< 1h	8/92 (8,7%)
H	23/92 (25%)
H	39/92 (41,3%)
>8 h	23/92 (25%)
**Millimeters of recession (mm)**	6,3 ± 1,04

Statistical analysis did not show statistically significant differences in terms of preoperative deviation, age at diplopia onset and surgical success between patients who spent more and less than 4 hours using electronic devices; nevertheless, the preoperative deviation was greater in patients who spent more than 4 hours at display screen (p>0,05). In addition, a negative correlation resulted between preoperative deviation and age at diagnosis (ρ = -0,261; p<0,05), whereas factors associated with surgical failure were older age at diplopia onset (p = 0,042), the longer delay between onset and diagnosis (p = 0,002), and a worse BCVA (p = 0,001) (Table 2).

**Table 2:** Clinical features of patients who underwent surgery.

	Total	Success	Failure	*p* ^a^
	(= 93)	(= 88)	(= 5)	
**Sex (M:F)**	29:64	29:59	0:5	
**Age at diplopia onset (y)**	35 ± 15.93	34,1 ± 15,58	50,8 ± 13.48	**0,042**
**Age at diagnosis (y)**	38,4 ± 16,5	37,1 ± 15,95	59,7 ± 11,22	**0,012**
**Interval between onset and diagnosis (y)**	3,33 ± 3,53	3,02 ± 3,15	8,86 ± 4,96	**0,02**
**Refraction in cycloplegia (D)**	-4,7 ± 2,5	-4,5 ± 2,5	-7,9 ± 4,9	0,201
**Deviation (PD)**				
Distance fixation	26,4 ± 7,2	26,8 ± 7	19,8 ± 7,2	0,073
Near fixation	25,7 ± 7,5	26 ± 7,4	19,8 ± 7,2	0,123
**Millimeters of recession (mm)**	6,3 ± 1	6,3 ± 1	6 ± 0,9	0,543
**BCVA (decimal)**	9,75 ± 0,83	9,82 ± 0.68	8,4 ± 1,81	**0,001**

^a^Test U of Mann-Whitney

Finally, the only statistically significant factor associated with surgical failure was the lower amount of recession. (p = 0,019).

**Fig 2:**
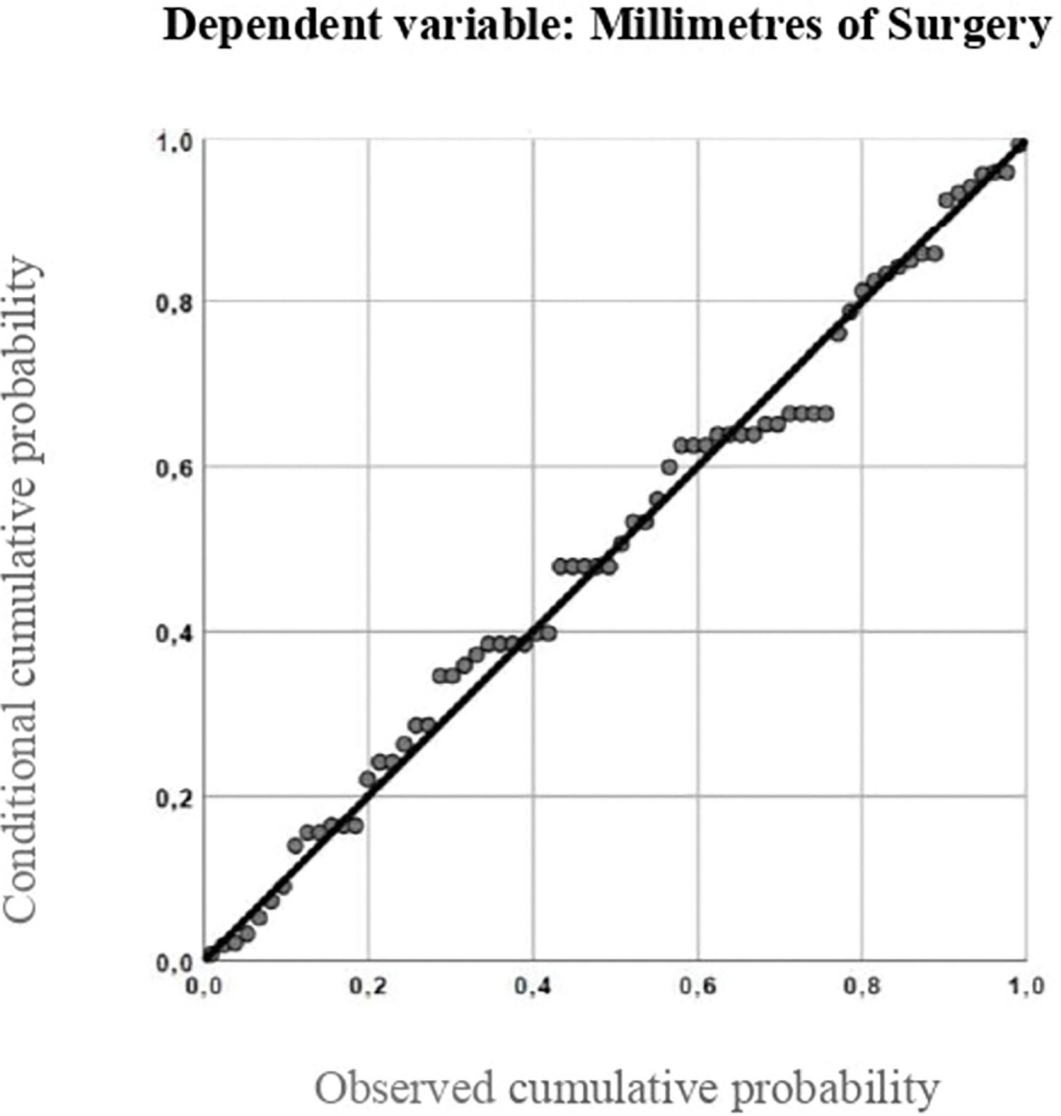
Simple linear regression model.

The simple linear regression model was esteemed, with values of R2 and adjusted R2 of 0,732 and 0,535, respectively (Fig 2). The formula obtained was y = 3.588 +0.105X, where y is the dependent variable (millimeters of surgery) and x is the independent variable (pre-operative angle of deviation at distance).

## Discussion

One of the main results emerged from the study, in contrast with other authors [8], was that a longer interval between diplopia onset and diagnosis influences fusional reserve after surgery with persistent diplopia in patients with a more long-lasting esodeviation compared to patients with a shorter time to diagnosis (3,02± 3,15 vs 8,86 ± 4,96 respectively (p = 0,02). Thus, ophthalmologists have to know the clinical features of this type of strabismus in order to avoid missing diagnosis and to delay the time of the treatment. The number of patients with a new diagnosis of type III AACE per year is increasing [9]. Institutional data of our hospital accounted for 1,2% (13/1092) of all first-visit strabismus patients from January 2013 to December 2015, 2,4% (27/1123) from January 2016 to December 2018, and 6,4% (57/896) from January 2019 to October 2021. We noticed a relationship with the exponential increase in the use of electronic devices (laptops, smartphones, tablets) for professional, educational, and recreational purposes, in accordance with other studies [10, 11]. In the present study, we observed that two-thirds of patients (66,3%) reported a history of intense use of electronic devices (> 4 h/day) at the time of diplopia onset. Electronic devices could lead to a closer lecture distance than the one generally employed during reading paper books [12]. Some authors suggested that this habit could determine a greater accommodative request that triggers an excessive convergence. Another hypothesis proposed by Turan et al is that closer lecture distance could increase the muscular tone of the medial rectus muscles and a weakening in lateral rectus muscles [13]. Lee et al. also speculated on a presumed dynamic activation of medial rectus muscles due to the short working distance during the use of mobile devices [10]. We hypothesized that the intensive use of near fixation at a close distance could reduce the ability to relax the convergence in the passage from near-to-distance fixation, and this would also explain the onset of diplopia at distance fixation for most of our patients (94%).

The study confirmed a possible role of myopia in the aetiology of type III AACE, though still not explained [14]. Bielschowsky’s hypothesis about the hypo-correction of myopia can be retracted since patients included in the study wear optic correction before the onset of diplopia. In the same way, we can exclude the role of an excess or a spasm of accommodation in patients with hyper-correction since it can also affect patients in whom the accommodation is poor. Moreover, we evaluated the deviation angle after a minimum of one month of full myopic correction, with and without glasses, observing no statistically significant difference (p>0,05).

Interestingly, almost all the patients (90,6%) did not remember the exact moment when symptoms began, and they usually reported transient episodes of diplopia at first, more and more frequently, until it became persistent. In particular, the diplopia started at distance fixation, when the visual field was reduced, such as during night driving or physical debilitation. For this reason, we assumed that it is appropriate to distinguish between the first two types of concomitant esotropia (Swan and Burian-Franceschetti), which are characterized by acute onset of esodeviation, from type III, in which diplopia occurs sub-acutely. This evidence could be explained through the phenomenon of *tenacious fusion* [15, 16], which is a compensatory mechanism used to restore ocular alignment by the fusional reserve. Consequently, in those situations in which these mechanisms are weakened, diplopia becomes evident, until becoming permanent when the fusional reserve runs out. Furthermore, the *tenacious fusion* phenomenon helps to explain the difficulty to measure the preoperative deviation. Indeed, patients can mask the esodeviation due to strong fusional reserve, which became evident during the cover test with the typical "eat-up prisms". Therefore, we suggest performing a very dissociative cover test, inviting the patient to alternatively fix a distance and a near stimulus to unmask the total deviation and thus avoid surgical hypo-corrections [3].

The mean age for esotropia onset was 35,07 ± 15,81 years, with a range of 9 to 71 years, which confirms that type III AACE is more common among young adults, but it does not rule out the involvement of children or the elderly. Recently, Spierer et al. proposed the definition of “acute concomitant esotropia of the adult” to describe a normosensorial esotropia, with a deviation that is the same for near and distance fixation, associated with myopia, but that involves only patients older than 16 years [17]. However, we believe this definition is incorrect because it includes only a subgroup of patients.

Acute concomitant esotropia can also be a clinical sign of intracranial tumor, Arnold-Chiari malformation or idiopatic intracranial hypertension [18]. Thus, evaluating other neurologic signs, such as nystagmus, muscular palsy, or lateral incomitance, is mandatory. Results confirmed that type III AACE appears to be not correlated to a neurologic cause. We suggest maintaining a high clinical suspicion and carrying out additional diagnostic tests in case of atypical features or low fusional amplitude [13].

Significant factors that predict surgical outcomes are age at diplopia onset, age at the diagnosis and the visual acuity at the baseline ophthalmological evaluation (p<0.05). Regarding the negative correlation between age and surgical success, we believe that elderly patients may present typical elements of both BE and age-related distance esotropia, such as peri-orbital tissue degeneration, which can negatively influence the surgical outcomes. On the other hand, we suggest that a better visual acuity at diagnosis ensures better sensorial fusional ability after surgery. Moreover, the deviation angle at diplopia onset was inversely correlated to age, and we hypothesized that the fusional reserve could reduce with age. Thus, lower esodeviation is sufficient to induce the development of diplopia in the elderly.

In patients who had yet undergone surgery we performed bilateral symmetric recession of medial rectus muscles. Based on our clinical practice and previous studies [19], AACEs require a wider surgery dosage than those reported in dose-response tables for esotropia [3]. Thanks to an augmented surgery of about one-third of the values reported in the Parks table, we accomplished good sensorial and motor outcomes, any hypo-correction, with a surgical success rate of 94,6%. Finally, we proposed a simple linear regression model based on data obtained on patients with surgical success with maintained ocular alignment during follow-up (Fig 2).

To our knowledge, this study included the largest number of patients with type III AACE, which aimed to describe clinical features and surgical outcomes. However, the retrospective nature of the study represents its major limit, mainly because it impedes the collection of some data. More studies will be necessary better to define fusional amplitude in patients with concomitant esotropia and to stabilize their role in the development of the strabismus and the maintenance of the alignment after the surgery. Moreover, further studies will be needed to better understand if the type and the size of different electronic devices could impact differently on the onset of strabismus.

## Conclusions

The results of our study showed that a prompt diagnosis and treatment are essential in the management of patients with type III AACE, since the gap between the onset of the diplopia and the diagnosis strongly influences the outcome after surgery. Moreover, our study showed that two-thirds of patients reported a history of intense use of electronic devices at the time of diplopia onset, suggesting a correlation between the use of electronic devices and the onset of diplopia. Finally, the main factors that determine good motor and sensorial results are a good visual acuity at the time of diagnosis, a younger age, and an augmented surgery dose.

## References

[1] Zhu M, Tang Y, Wang Z, Shen T, Qiu X, Yan J, et al. Clinical characteristics and risk factors of acute acquired concomitant esotropia in last 5 years: a retrospective case–control study. Eye. 2022;1–5. doi: 10.1038/s41433-022-01939-1 35075284 PMC9873604

[2] Burian HM, Miller JE. Comitant convergent strabismus with acute onset. Am J Ophthalmol. 1958;45(4 Pt 2):55–64. doi: 10.1016/0002-9394(58)90223-x 13520873

[3] Roda M, Pellegrini M, Rosti A, Fresina M, Schiavi C. Augmented bimedial rectus muscles recession in acute acquired concomitant esotropia associated with myopia. Can J Ophthalmol. 2021 Jun;56(3):166–70. doi: 10.1016/j.jcjo.2020.10.006 33160920

[4] Lekskul A, Chotkajornkiat N, Wuthisiri W, Tangtammaruk P. Acute Acquired Comitant Esotropia: Etiology, Clinical Course, and Management. Clin Ophthalmol. 2021;15:1567. doi: 10.2147/OPTH.S307951 33883873 PMC8055253

[5] Neena R, Remya S, Anantharaman G. Acute acquired comitant esotropia precipitated by excessive near work during the COVID-19-induced home confinement. Indian J Ophthalmol. 2022;70(4):1359. doi: 10.4103/ijo.IJO_2813_21 35326055 PMC9240503

[6] Van Hoolst E, Beelen L, De Clerck I, Petit L, Balikova I, Casteels I, et al. Association between near viewing and acute acquired esotropia in children during tablet and smartphone use. Strabismus. 2022; doi: 10.1080/09273972.2022.2046113 35291920

[7] Mohan A, Sen P, Mujumdar D, Shah C, Jain E. Series of cases of acute acquired comitant esotropia in children associated with excessive online classes on smartphone during COVID-19 pandemic; digital eye strain among kids (DESK) study-3. Strabismus. 2021;29(3):163–7. doi: 10.1080/09273972.2021.1948072 34223812

[8] Fu T, Wang J, Levin M, Xi P, Li D, Li J. Clinical features of acute acquired comitant esotropia in the Chinese populations. Medicine (Baltimore). 2017 Nov;96(46). doi: 10.1097/MD.0000000000008528 29145257 PMC5704802

[9] Yilmaz PT, Fatihoglu ÖU, Sener EC. Acquired Comitant Esotropia in Children and Young Adults: Clinical Characteristics, Surgical Outcomes, and Association With Presumed Intensive Near Work With Digital Displays. J Pediatr Ophthalmol Strabismus. 2020 Aug;57(4):251–6. doi: 10.3928/01913913-20200422-02 32687210

[10] Lee HS, Park SW, Heo H. Acute acquired comitant esotropia related to excessive Smartphone use. BMC Ophthalmol. 2016 Apr 9;16(1). doi: 10.1186/s12886-016-0213-5 27061181 PMC4826517

[11] Mehta A, Greensher JE, Dahl GJ, Miller KE. Acute Onset Esotropia From Excessive Smartphone Use in a Teenager. J Pediatr Ophthalmol Strabismus. 2018 Dec 19;55(6):E42–4. 30571837 10.3928/01913913-20181017-01

[12] Bababekova Y, Rosenfield M, Hue JE, Huang RR. Font size and viewing distance of handheld smart phones. Optom Vis Sci. 2011 Jul;88(7):795–7. doi: 10.1097/OPX.0b013e3182198792 21499163

[13] Turan KE, Kansu T. Acute Acquired Comitant Esotropia in Adults: Is It Neurologic or Not? J Ophthalmol. 2016;2016. 10.1155/2016/2856128 PMC5149673 28018672

[14] Sturm V, Menke MN, Knecht PB, Schöffler C. Long-term follow-up of children with acute acquired concomitant esotropia. J AAPOS. 2011 Aug;15(4):317–20. doi: 10.1016/j.jaapos.2011.03.018 21907110

[15] Brodsky MC, Jung J. Intermittent Exotropia and Accommodative Esotropia: Distinct Disorders or Two Ends of a Spectrum? Ophthalmology. 2015 Aug 1;122(8):1543–6. doi: 10.1016/j.ophtha.2015.03.004 26210597

[16] Kushner BJ, Morton G V. Distance/near differences in intermittent exotropia. Arch Ophthalmol. 1998;116(4):478–86. doi: 10.1001/archopht.116.4.478 9565045

[17] Spierer A. Acute concomitant esotropia of adulthood. Ophthalmology. 2003 May 1;110(5):1053–6. doi: 10.1016/S0161-6420(03)00102-7 12750113

[18] Hoyt CS, Good W V. Acute onset concomitant esotropia: When is it a sign of serious neurological disease? Br J Ophthalmol. 1995;79(5):498–501. doi: 10.1136/bjo.79.5.498 7612566 PMC505143

[19] Archer SM. Why strabismus surgery works: the legend of the dose-response curve. J AAPOS Off Publ Am Assoc Pediatr Ophthalmol Strabismus. 2018 Feb 1;22(1):1.e1–1.e6. doi: 10.1016/j.jaapos.2017.12.001 29288836

