# Correction: Epidemiology, clinical features, and surgical outcomes of acute acquired concomitant esotropia associated with myopia

**DOI:** 10.1371/journal.pone.0308312

**Published:** 2024-07-31

**Authors:** The PLOS ONE Editors

## Notice of republication

This article [1] was republished on April 22, 2024, to address an issue identified post-publication. An updated version of S1 File is provided with this notice. Please download this article again to view the correct version.

## Supporting information

S1 File Underlying data. (XLSX)
